# Phenotypic Diversity Assessment of Okra (*Abelmoschus Esculentus* (L.) Moench) Genotypes in Ethiopia Using Multivariate Analysis

**DOI:** 10.1155/2022/3306793

**Published:** 2022-03-12

**Authors:** Jemal Mohammed, Wassu Mohammed, Eleni Shiferaw

**Affiliations:** ^1^Crop and Horticulture Biodiversity Directorate, Ethiopian Biodiversity Institute, Addis Ababa, Ethiopia; ^2^School of Plant Science, Haramaya University, Dire Dawa, Ethiopia

## Abstract

Okra is a minor crop that has not gained research attention in Ethiopia. Characterization of such underutilized crops has important implications for their utilization. Thus, this study was conducted to assess the genetic diversity of okra genotypes in Ethiopia using agromorphological and biochemical markers. Thirty-six okra genotypes were evaluated for 29 agromorphological and biochemical traits. The results of the analysis of variance showed significant differences among genotypes for most of the traits, except for the number of flower epicalyx and fruit diameter. Results of the principal component analysis indicated that the first eight principal component axes accounted for 3.83 to 30.54% and 82.44% of the total variability. Genetic distances estimated by Euclidean distances from 27 traits ranged from 3.55 to 14.49. The 36 genotypes were grouped into four distinct clusters from the Euclidean distance matrix using the unweighted pair group method with arithmetic mean (UPGMA). The first cluster contained 24 (66.66%) genotypes, and the second cluster contained 10 (27.77%) of the genotypes. This study showed the presence of considerable genetic variation among the genotypes for most of the traits, including fruit yield, seed yield, and nutrient content of seeds, indicating the possibility of using these genotypes to develop okra varieties with high fruit-yielding and good nutritional content.

## 1. Introduction

Today, the world's food supply is based on a small number of crop species, mostly major cereals (wheat, rice, and maize) leaving an abundance of genetic resources and potentially beneficial traits neglected [1]. In the face of climate change, utilizing the vast pool of minor and underutilized crop species would provide a more varied agricultural system and food sources, ensuring food and nutrition security problems. Underutilized crops play a significant role in ensuring food security, nutrition, and income generation for resource-poor farmers and consumers, especially in the developing world [2].

Climate change may increase the relevance of plant species that were previously underutilized or thought to be of minor importance [1]. One approach to maintaining a good fit between crops and environmental challenges because of climate change is to use underutilized (minor, orphan, or neglected) crops and their wild relatives. Okra is among the most underutilized crops cultivated in the southwestern and western parts of Ethiopia [3–5]. The characterization of okra genotypes existing in the country would contribute to developing varieties that could thrive in extreme climatic conditions and would allow further utilization of the crop for enhancing food security.

The value of germplasm collections depends on their diversity, and crop improvement prominently relies on existing genetic variation [6, 7]. Shujaat et al. [8] suggested that genetic variation is an important feature to achieve the diversified goals of plant breeding, including higher and quality yield, resistance to diseases, and wider adaptations.

The pattern and level of genetic diversity in a given gene pool can be measured in terms of genetic distance, which is a measurement of average genetic divergence between genotypes or populations [9]. Regardless of the dataset (morphological, biochemical, or molecular marker data), multivariate analytical procedures that simultaneously make several measurements on each individual under examination are frequently utilized in genetic diversity studies [10]. This study aimed to assess the phenotypic and biochemical diversity of Ethiopian landrace okra genotypes along with exotic commercial varieties using multivariate analysis for further utilization of the crop and contribute to ensuring food security and alleviating malnutrition.

## 2. Materials and Methods

### 2.1. Description of the Study Site

The field experiment was conducted at the Melkassa Agricultural Research Center, Ethiopia, during the 2018 main cropping season (rainy season). Melkassa is located at 8°24′59.20″ N latitude and 39°19′15.19″ E longitude, with an altitude of 1,548 m above sea level [11]. The biochemical contents were determined at the Ethiopian Biodiversity Institute Nutrition Laboratory (total ash and crude fat), the Debre Ziet Agricultural Research Center (crude fiber), and the Melkassa Agricultural Research Center (total protein).

### 2.2. Experimental Materials and Design

Thirty-six okra genotypes of 24 landrace accessions (collected by the Ethiopian Biodiversity Institute from different okra growing regions of Ethiopia), three genotypes (from the Humera Agricultural Research Center), and nine exotic commercial varieties (eight from India and one from the USA) were used in this study. The 36 genotypes were planted in a 6 × 6 simple lattice design. Three seeds per hill were sown and thinned to one plant per hill when plants reached the 3–4 leaf stage.

### 2.3. Data Collection

Data were collected for phenology traits (days to 50% emergence, days to first flowering, days to 50% flowering, and days to 90% maturity), growth and yield-related traits (plant height, stem diameter, number of primary branches per stem, number of internodes, internodes length, leaf length, leaf width, number flower of epicalyxes, peduncle length, fruit length, fruit diameter, average fruit weight, number of tender fruits per plant, number of mature pod per plant, number of ridges on fruit, fruit yield per plant, fruit yield per hectare, number of seeds per pod, hundred seed weight, seed yield per plant, and seed yield per hectare), and biochemical content of the seed (total ash, total fat, crude fiber, and total protein). Phenology and growth-related traits' data were recorded according to the IPGRI [12] descriptor list developed for okra.

#### 2.3.1. Total Ash

Total ash was determined following the method of AOAC [13] using the gravimetric method. Crucible was cleaned, dried, and ignited at 550°C for 1 hour and weighed (m1). The flour sample (3 g) weighed (m2) and dried at 120°C for 1 hour. Then, the dried sample was carbonized over a blue flame and ignited in a muffle furnace at 550°C until ashing was complete (over 12 hrs). After being ignited, the sample was cooled to ambient temperature and was weighed (m3). Finally, the total ash content was calculated as follows:

(1) $$\begin{array}{c} \text{Ash }\left(\%\right)=\left(\frac{m3-m1}{m2-m1}\right)*100, \end{array}$$

where m1 is the mass of crucible (g), m2 is sample mass with crucible (g), and m3 is the final mass of sample with crucible (g).

#### 2.3.2. Crude Fat

The crude fat content of okra seed was determined by the Soxhlet extraction method according to the AOAC [13]. The flour sample (3 g) was weighed and added into a thimble. The thimble with the sample was placed in a 50 ml beaker and dried in an oven for 2 hours at 110°C. A 150–250 ml dried beaker was weighed and rinsed several times with petroleum ether. The sample contained in the thimble was extracted with petroleum ether in a Soxhlet extraction apparatus for 6–8 hours. After extraction is completed, the extracted fat was transferred into a preweighed beaker (*M*_i_). The beaker with the extracted fat was placed in a fume hood to evaporate the solvent on a steam bath unit no odor of the solvent is detectable. Then, the beaker with contents was removed, cooled in a desiccator, and weighed (*M*_f_). The amount of fat in flour was calculated by using the following formula:

(2) $$\begin{array}{c} \text{Fat }\left(\%\right)=\left(\frac{Mf-Mi}{M}\right)*100, \end{array}$$

where *M*_f_ is the dried mass of the fat with beaker (g), *M*_i_ is the mass of beaker (g), and *M* is the sample mass (g).

#### 2.3.3. Crude Fiber

The crude fiber was determined according to the AOAC [13]. Ground sample (3 g) was weighed (*m*_1_), placed in 500 ml beaker, digested with 1.25% sulfuric acid, and washed with water and was further digested with 1.25% sodium hydroxide and filtered in course porous (75 *μ*m) crucible in apparatus at a vacuum of about 25 mm. The residual left after refluxing was washed again with 1.25% sulfuric acid at near boiling point. Then, the residual was dried at 110°C overnight, cooled in a desiccator, and weighed (*m*_2_). After being dried, the sample was ashed at 550°C until the ashing was complete, cooled in a desiccator, and weighed again (m3). The total crude fiber was expressed in percentages as follows:

(3) $$\begin{array}{c} \text{Fiber }\left(\%\right)=\left(\frac{m2-m3}{m1}\right)*100, \end{array}$$

where m1 is a mass of sample (g), m2 is mass of sample with crucible before ashing (g), and m3 is mass of sample with crucible after ashing (g).

#### 2.3.4. Total Protein

A dried and grounded sample was taken (0.5 g) and added into a *Kjeldahl* digestion flask. One gram of catalyst (Na_2_SO_4_ mixed with anhydrous CuSO_4_ in a ratio of 10 : 1) and 5 ml of concentrated H_2_SO_4_ were added into the digestion flask. Then, using a digester, the mixed sample was digested at 350°C for about two hours until the sample was completely digested. Then, the flask was removed from the digester and allowed to cool and the digested sample was diluted by adding 30 ml of distilled water. Then, 25 ml concentrated 40% NaOH was added into the digestion flask to neutralize the acid and make the solution slightly alkaline. The contents were immediately distilled by inserting the digestion tube line into the receiver flask that contained 25 ml of 4% boric acid solution and about 150 ml of distillate collected. Then, the distillate was titrated by a standard acid (0.1 N HCl). The percentage of crude protein was calculated by multiplying the nitrogen percentage by the conversion factor (6.25) [13].

(4) $$\begin{array}{c} \text{Nitrogen }\left(\%\right)=\;\frac{V\text{ HCI }A\;-\;V\text{ HCI }B\;*\;N\text{ HCI }*\;0.014}{W\text{ sample}}*100, \end{array}$$

where *V* = volume of standard acid used for titration of sample (A) and blank sample (B), *N* = normality of standard acid used for titration (0.1 N HCl), 0.014 is the molecular weight of nitrogen, and *W* = weight sample taken for digestion, on a dry basis.

(5) $$\begin{array}{c} \text{Protein }\left(\%\right)=6.25*\%\text{ Nitrogen}. \end{array}$$

### 2.4. Analysis of Variance

The quantitative field data were subjected to analysis of variance (ANOVA) and computed with R statistical software agricolae package [14]. The biochemical traits were analyzed following the CRD (completely randomized design) procedure. The traits that exhibited significant mean squares in ANOVA were further subjected to multivariate analysis.

### 2.5. Principal Component Analysis

Principal component analysis (PCA) was computed to find out the traits, which accounted more for the total variation. The data were standardized to mean zero and variance of one before computing principal component analysis to avoid differences in measurement scales. The principal component based on the correlation matrix was calculated using the R statistical software FactoMineR package [15].

### 2.6. Euclidean Distance and Clustering of Genotypes

Euclidean distance (ED) was computed from quantitative after subtracting the mean value and dividing it by the standard deviation as established by Sneath and Sokal [16]. R statistical software factoextra package [17] was used for the analysis of distance matrix and constructing dendrogram. The dendrogram was constructed based on the unweighted pair group method with arithmetic mean (UPGMA) from the distance matrix of phenotypic traits.

## 3. Results and Discussion

### 3.1. Analysis of Variance

The results of the analysis of variance for phenology, growth, yield-related traits, and biochemical traits showed a significant (*P* < 0.05) difference. However, the genotypes exhibited a nonsignificant difference for the number of flower epicalyx and fruit diameter (Tables 1 and 2).

### 3.2. Principal Component Analysis

The result of principal component analysis for 27 quantitative traits is presented in Table 3. With eigenvalues ranging from 1.033 to 8.247, the principal component analysis resulted in eight principal components (PC1 to PC8). The eight principal components each accounted for a different percentage of the total variance, ranging from 3.83 to 30.54%, for a total variance of 82.44%. The PCs with an eigenvalue of <1 were ignored due to Gutten's lower bound principle that eigenvalues <1 should be ignored. The first principal component (PC1) contributed to most of the variation (30.54%), followed by PC2, PC3, and PC4, which contributed 14.11%, 10.87%, and 6.98%, of the variation respectively, and the first four PCs accounted for 62.51% of the total variation.

A similar result on okra was reported by Muluken et al. [18] in which the first three principal components PC1, PC2, and PC3, with values of 32.4%, 16.7%, and 8.2%, respectively, contributed more to the total of 57.3% variation. Amoatey et al. [19] reported the first, second, and third principal components with values of 32.44%, 19.78%, and 9.68% of the total genetic variation, respectively. Ahiakpa [20] also reported that the first principal component (PC1) was (32.44%) the major contributor for variance in okra genotypes.

Within the PC1, traits with the largest values closer to one influence the cluster more than traits with lower absolute values closer to zero [21]. Hence, the differentiation of the genotypes into different clusters was because of the cumulative effect of several traits rather than the large contribution of a few traits. In this regard, stem diameter (7.41%), fruit yield per hectare (8.03%), leaf width (7.56%), fruit yield per plant (7.87%), leaf length (7.11%), peduncle length (6.75), seed yield per plant (6.75%), and seed yield per hectare (6.75%) had relatively higher contributions to PC1. This indicates that these traits were responsible for the differentiation of the clusters and had a greater contribution to the total diversity. In PC2, days to 50% flowering (18.58%), days to first flowering (16.87%), date of maturity (14.76%), and the number of mature pods (8.12%) had more contribution, whereas fruit length, fresh fruit weight, and ash content had relatively more contribution in PC3 (Table 3).

**Figure 1:**
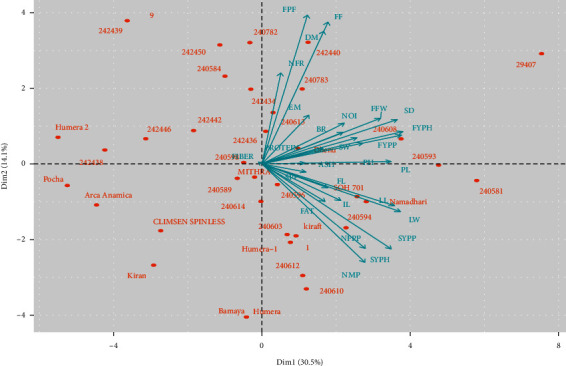
Biplot (axes PC1 and PC2) of 27 quantitative traits of 36 okra genotypes.

A biplot was performed based on the first two PCs (Figure 1). The genotypes and quantitative traits were shown on a biplot to visualize their associations. The first and the second PC biplots explained 44.66% of the total variability among the genotypes, displaying that stem diameter, leaf length, and leaf width, fruit yield per plant, fruit yield per hectare, seed yield per plant, and seed yield per hectare were considered the most discriminating traits.

The genotypes positioned on the right top quadrant were characterized by late maturity, high fresh fruit weight, much fruit ridges, and high stem diameter. The genotypes depicted in the bottom right quadrant had the highest seed yield, number of fruits per plant, number of mature pods, and longest, and widest leaf. The genotypes distributed around the origin had similar genetic characteristics, while the genotypes that were found far from the origin are considered unrelated genotypes (Figure 1). Therefore, these divergent genotypes could be used as potential parents for successful hybridization to develop heterotic groups in the okra-breeding program.

**Figure 2:**
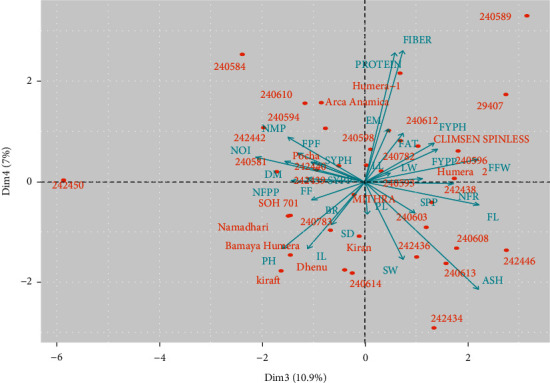
Biplot (axes PC3 and PC4) of 27 quantitative traits of 36 okra genotypes.

PC3 and PC4 biplots are presented in Figure 2. These two PCs accounted for 17.85% of the total variability among genotypes, showing that ash content, and total protein content, crude fiber content, fruit length, and fresh fruit weight were the most contributing traits. PC3 and PC4 biplots provided information regarding the similarities and the pattern of differences among the okra genotypes and the association between traits. Genotypes were distributed in all four quadrants on the axes, indicating the presence of wide genetic variability for the traits studied. Overlapped accessions and accessions closer to each other on the axes had similar genetic makeup. However, genotypes that are apart from each other could be considered genetically distinct. Genotypes positioned in the top right quadrant were characterized by high-seed protein and fiber content. The top left quadrant consists of the okra genotypes that are closely related and have a high number of internode and a high number of mature pods. Genotypes found on the right bottom quadrant exhibited the highest seed ash content.

### 3.3. Cluster Analysis

**Figure 3:**
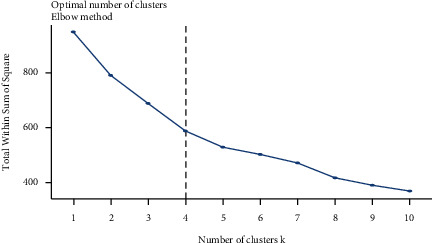
Determination of the optimum number of clusters K using total within sum of square (WSS) method.

The optimum number of clusters was determined by the total within sum of square (WSS) (elbow method) using R statistical software version 3.6.3 (Figure 3). A dendrogram was constructed based on the unweighted pair group method with arithmetic mean (average) from the distance matrix of phenotypic traits.

**Figure 4:**
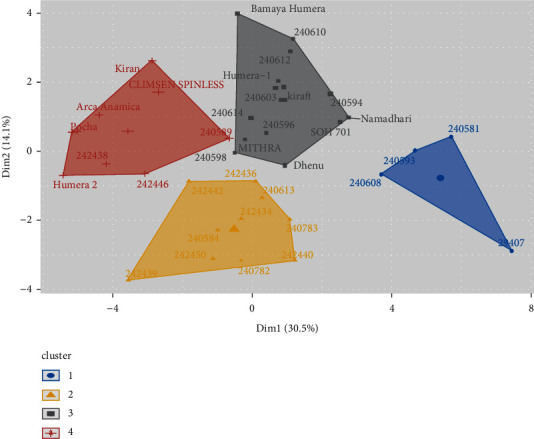
Scatter plot constructed based on PC1 and PC2 for 27 quantitative traits.

The distances of all possible pairs of the 36 okra genotypes from 27 quantitative traits were estimated by Euclidean distance. The distances between okra genotypes ranged from 3.55 to 14.49 with a mean, standard deviation, and coefficient of variation of 7.12, 1.80, and 25.25%, respectively. The highest genetic distance (Euclidean distance) was computed between 29407 and Humera 2 (14.49), whereas the lowest genetic distance was estimated between Dhenu and Mithra (3.55) (Figure 4).

Based on PC axes 1 and 2, a scatter plot was constructed for four clusters (Figure 4). The plot showed that the genotypes that have similar genetic makeup were grouped in a cluster (near to overlap), and those genotypes that have different genetics were positioned in the opposite corner of the scatter plot.

Generally, the Euclidean distances measured among the introduced varieties were lower than the genetic distances among genotypes collected from Ethiopia. This showed that there is a higher chance of improving fruit yield and seed-related traits through the selection and/or hybridization of okra genotypes collected from different okra growing regions of Ethiopia.

By characterizing 24 Ethiopian okra genotypes, Fozia [22] reported Euclidean distance that ranged from 1.96 to 11.36 with a mean, standard deviation, and coefficient of variation of 5.85, 1.97, and 33.75%, respectively. The same study also reported that introduced (Indian) varieties had lower (1.96 to 10.01) genetic distances than Ethiopia's okra collection, which ranged from (2.07 to 11.36). Muluken et al. [18] reported that Ethiopian okra collections exhibited a wider genetic distance than exotic varieties. Anteneh [23] estimated the genetic distances of all possible pairs of 25 okra genotypes and reported that the highest genetic distances were observed between okra collections from Ethiopia and introduced commercial varieties from other countries, while the lowest genetic distance was estimated between introduced commercial varieties.

The extent of diversity present between genotypes determines the extent of improvement gained through selection and hybridization. The more distant the two genotypes are, the greater the probability of improvement through selection and hybridization. Mihretu et al. [3] also reported the presence of considerable genetic distance among okra collections from Gambela regional state, which is one of the okra-growing regions in Ethiopia.

**Figure 5:**
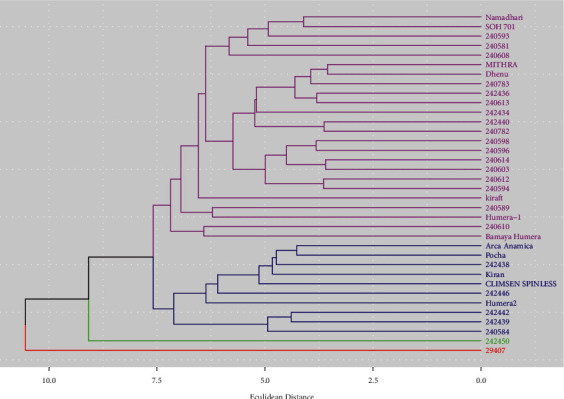
Dendrogram illustrating dissimilarity of 36 okra genotypes by an average method of clustering method from Euclidean distance matrix based on 27 traits.

Clustering of genotypes based on Euclidean distances revealed four major clusters. The number and names of genotypes in each cluster along with their collection origin are presented in Table 4. Cluster I consists of the majority of the genotypes, which accounted for 24 (66.66%) of the genotypes. Cluster II contains 10 (27.77%) genotypes, while clusters III and IV each contain only single genotypes (Table 4, Figure 5).

Genotypes clustered in cluster I and cluster II were early maturing, while the two genotypes positioned in cluster III and cluster IV were late-maturing genotypes (Table 5). Therefore, genotypes found in cluster I and cluster II could be used for okra production in areas characterized by the low amount of rainfall. These genotypes could also be used by breeders for developing varieties suitable for drought-prone areas. On the contrary, the two genotypes found in clusters III and IV could be used for areas that have a long rainfall season. The highest mean of fruit yield was measured from the genotype (29407), which is found in cluster IV. This genotype also had the highest seed protein content. This genotype could be used for further evaluation to identify genotypes with high fruit yield and desirable nutrient contents.

## 4. Conclusions

The results showed the presence of considerable genetic diversity for the studied morphological and biochemical traits. This variation could be exploited to develop varieties with different desirable agronomic traits like early maturing, high yield, and good nutrient content through either selection and/or hybridization using the okra genotypes collected and conserved in Ethiopia.

In addition, the study revealed the potential of the landrace okra genotypes as sources of nutrients. This indicates the importance of neglected crops that could be utilized for ensuring food security and alleviating malnutrition in developing countries like Ethiopia, where malnutrition is a widespread problem. It is also recommended to extend the research in okra to include micronutrient content analysis, molecular diversity study, and sequencing okra genotypes to identify important agronomic and biochemical traits and to characterize the genes responsible for the traits.

## Figures and Tables

**Table 1:** Mean square from analysis variance following simple lattice design for 25 quantitative traits of 36 okra genotypes evaluated at Melkassa in 2018.

Traits	Rep. (1)	Blocks within rep. (Adj.) (10)	Treatments (uadj.) (35)	Intrablock error (25)	Total (71)	CV (%)
Days to 50% emergence	1.6806	0.1139	0.2964^*∗*^	0.1472	0.2377	5.03
Days to first flowering	0.1250	2.9917	22.2679^*∗∗*^	2.4183	12.251	2.85
Days to 50% flowering	7.3472	1.2306	22.6806^*∗∗*^	3.1939	12.582	3.03
Days to maturity	24.500	3.8000	25.1000^*∗∗*^	4.2600	14.753	2.60
Stem diameter (cm)	0.1184	0.1539	0.1401^*∗∗*^	0.0534	0.1112	13.11
Plant height (cm)	1773.6	459.73	1153.51^*∗∗*^	253.55	747.64	13.94
Number of branches	0.5322	0.1484	1.9838^*∗∗*^	0.1464	1.0579	17.17
Number of internodes	1.6200	4.0694	6.9937^*∗*^	3.3589	5.2261	12.35
Internode length (cm)	3.5778	0.9520	5.0346^*∗∗*^	0.6670	2.9012	15.32
Leaf length (cm)	0.8712	0.8712	8.0159^*∗*^	3.6743	6.0443	13.30
Leaf width (cm)	13.589	16.2971	21.1354^*∗∗*^	8.7260	15.978	14.64
Number of epicalyx	0.0383	1.8665	2.4630 ns	1.2649	1.9200	10.14
Peduncle length (cm)	0.0168	0.0772	0.2807^*∗∗*^	0.0402	0.1636	10.12
Fruit length (cm)	16.093	6.5258	13.6482^*∗∗*^	1.6781	8.4647	8.38
Fruit diameter (cm)	0.0621	0.2527	0.2067 ns	0.1186	0.1801	12.08
Number of fruit ridge	0.5270	0.6405	1.6811^*∗∗*^	0.4092	1.0704	9.06
Fresh fruit weight (g)	118.49	32.4883	190.64^*∗∗*^	30.137	110.83	15.78
Number of fruits per plant	3.5289	8.1578	35.4541^*∗∗*^	6.6569	21.020	9.42
Number of mature pods	1.0035	3.6576	19.8511^*∗∗*^	2.7918	11.298	18.38
Fruit yield per plant (kg)	0.1663	0.0737	0.1513^*∗∗*^	0.0455	0.1033	22.22
Fruit yield (ton ha^−1^)	84.990	28.0188	63.921^*∗∗*^	22.096	44.434	23.43
Number of seeds per pod	257.42	74.1880	365.41^*∗∗*^	87.239	224.92	9.66
Hundred seeds' weight (g)	0.4868	0.1993	0.6721^*∗∗*^	0.2004	0.4368	6.77
Seed yield per plant (g)	595.09	335.18	953.98^*∗∗*^	170.33	585.85	22.29
Seed yield per hectare (kg)	258286	145475	414055^*∗∗*^	73925.9	254269	22.29

^∗∗, ∗^ and ns, significant at *P* < 0.01, *P* < 0.05, and nonsignificant, respectively. Rep = replication, Adj = adjusted, Uadj = unadjusted, CV (%) = coefficient of variation in percent. The number in parenthesis in each source of variation represents the degree of freedom.

**Table 2:** Mean square from analysis variance for four seed biochemical contents of 36 okra genotypes evaluated at Melkassa in 2018.

Source of variation	DF	Total ash (%)	Crude fat (%)	Crude fiber (%)	Total protein (%)
Genotypes	35	0.0948^*∗∗*^	1.6531^*∗∗*^	21.52^*∗∗*^	52.53^*∗∗*^
Error	36	0.0038	0.5031	0.4640	0.0538
CV (%)		1.44	4.62	2.40	2.03

^∗∗^, significant at P<0.01, DF = degree of freedom, and CV (%) = coefficient of variation in percent.

**Table 3:** Factor loading of the first eight principal components for 27 quantitative traits of 36 okra genotypes.

Traits	Principal components							
	PC1	PC2	PC3	PC4	PC5	PC6	PC7	PC8
Days to 50% emergence	0.27	0.28	0.11	0.26	−0.48	0.13	0.31	−0.41
Days to first flowering	0.38	0.80	−0.28	0.02	−0.25	−0.09	−0.02	0.06
Days to 50% flowering	0.26	0.84	−0.32	0.14	−0.21	−0.11	0.02	0.09
Days to maturity	0.36	0.75	−0.39	0.10	−0.31	−0.13	0.00	0.07
Stem diameter (cm)	0.78	0.25	−0.16	−0.21	0.18	0.14	−0.03	−0.02
Plant height (cm)	0.58	0.12	−0.40	−0.33	0.34	0.08	0.10	0.23
Number of branches	0.47	0.18	−0.26	−0.09	0.47	0.08	0.58	−0.15
Number of internodes	0.48	0.23	−0.53	0.12	0.43	0.15	−0.19	−0.14
Internode length (cm)	0.46	−0.21	−0.28	−0.33	−0.31	−0.19	0.16	0.31
Leaf length (cm)	0.77	−0.24	0.12	0.04	0.18	−0.16	0.28	−0.24
Leaf width (cm)	0.80	−0.27	0.28	0.02	0.04	0.09	0.18	−0.07
Peduncle length (cm)	0.75	0.01	0.01	−0.16	−0.30	0.13	−0.03	−0.16
Fruit length (cm)	0.38	−0.14	0.55	−0.11	−0.40	−0.28	−0.01	0.03
Number of fruit ridge	0.11	0.51	0.43	−0.01	−0.12	0.39	−0.15	−0.25
Fresh fruit weight (g)	0.68	0.26	0.55	0.11	0.25	−0.03	−0.16	0.04
Number of fruit per plant	0.60	−0.48	−0.35	0.01	−0.16	−0.24	0.03	−0.21
Number of mature pod	0.59	−0.56	−0.37	0.22	−0.11	−0.10	−0.23	−0.14
Fruit yield per plant (kg)	0.81	0.16	0.35	0.16	0.21	−0.17	−0.19	−0.02
Fruit yield (t ha^−1^)	0.81	0.18	0.33	0.19	0.18	−0.16	−0.19	−0.01
Number of seeds per pod	0.25	−0.05	0.24	−0.15	−0.16	0.80	0.12	0.24
Hundred seeds' weight (g)	0.55	0.15	0.18	−0.38	−0.09	−0.12	−0.02	0.39
Seed yield per plant (g)	0.75	−0.48	−0.26	0.10	−0.13	0.21	−0.18	0.09
Seed yield (kg ha^−1^)	0.75	−0.48	−0.26	0.10	−0.13	0.21	−0.18	0.09
Total ash (%)	0.35	0.09	0.55	−0.53	0.16	−0.18	−0.02	−0.06
Crude fat (%)	0.36	−0.21	0.18	0.24	−0.30	−0.04	0.44	0.17
Crude fiber (%)	−0.02	0.01	0.18	0.65	0.29	−0.09	0.31	0.34
Total protein (%)	0.25	0.01	0.15	0.64	−0.01	0.10	−0.14	0.25
**Eigenvalue**	**8.25**	**3.81**	**2.94**	**1.88**	**1.83**	**1.34**	**1.18**	**1.03**
**Contribution (%)**	**30.54**	**14.11**	**10.87**	**6.98**	**6.77**	**4.97**	**4.36**	**3.82**
**Cumulative variance (%)**	**30.54**	**44.66**	**55.53**	**62.51**	**69.28**	**74.25**	**78.61**	**82.44**

PC1 to PC8 represent the first principle component to the eighth principal component.

**Table 4:** Clusters, number of accessions, name of genotypes, and collection origin of 36 okra genotypes.

Clusters	Number of genotypes	Genotypes	Collection origin (district)
I	24	Namadhari, SOH 701, 240593, 240581, 240608, Mithra, Dhenu, 240783, 242436, 240613, 242434, 242440, 240782, 240596, 240598, 240614, 240603, 240612, 240594, Kraft, 242589, Humera 1, 240610, Bamaya Humera	Southwestern Ethiopia (Gog), southwestern Ethiopia (Gambela), southwestern Ethiopia (Itang), northwestern Ethiopia (Dangur), western Ethiopia (Asosa), northwestern Ethiopia (Metema) southwestern Ethiopia (Akobo)
II	10	Arka Anamica, Pocha, 242438, Kiran, Clemson Spinless, 242446, Humera 2, 242442, 242439, 240584	Indian commercial variety, American commercial variety, western Ethiopia (Asosa), southwestern Ethiopia (Akobo)
III	1	242450	Western Ethiopia (Asosa
IV	1	29407	Western Ethiopia (Kurmuk)

**Table 5:** Cluster mean value for 27 quantitative traits of 36 okra genotypes.

Traits	Clusters			
	I	II	III	IV
Days to 50% emergence	7.63	7.60	7.50	8.00
Days to first flowering	54.46	53.60	59.00	61.50
Days to 50% flowering	58.48	58.50	65.00	65.00
Days to maturity	79.08	78.30	85.00	87.00
Stem diameter (cm)	1.84	1.52	1.92	2.27
Plant height (cm)	120.65	93.84	162.03	113.92
Number of branches	2.32	1.75	4.13	3.00
Number of internodes	15.12	13.47	20.93	15.47
Internode length (cm)	5.69	4.47	4.97	5.85
Leaf length (cm)	15.02	12.40	15.17	19.00
Leaf width (cm)	21.28	17.37	14.84	27.10
Peduncle length (cm)	2.10	1.64	1.75	2.82
Fruit length (cm)	15.89	14.35	8.25	22.87
Number of fruit ridge	7.14	6.77	7.00	7.91
Fresh fruit weight (g)	37.40	27.09	20.82	62.41
Number of fruit per plant	28.55	24.02	29.54	31.46
Number of mature pods	10.04	6.55	9.50	11.25
Fruit yield per plant (kg)	1.04	0.69	0.77	1.87
Fruit yield (ton ha^−1^)	21.74	14.58	15.94	38.86
Number of seeds per pod	100.11	92.52	73.40	77.60
Hundred seeds' weight (g)	6.76	6.28	5.63	7.37
Seed yield per plant (g)	67.38	38.52	39.22	65.22
Seed yield per hectare (kg)	1403.85	802.55	817.13	1358.82
Total ash (%)	4.35	4.18	3.96	4.63
Crude fat (%)	15.58	14.88	13.14	16.59
Crude fiber (%)	29.94	31.29	28.99	32.72
Total protein (%)	10.71	11.37	6.56	20.94

## Data Availability

The raw data and additional information could be made available from the corresponding author upon request.
